# Gamma Band Neural Synchronization Deficits for Auditory Steady State Responses in Bipolar Disorder Patients

**DOI:** 10.1371/journal.pone.0039955

**Published:** 2012-07-05

**Authors:** Yuko Oda, Toshiaki Onitsuka, Rikako Tsuchimoto, Shogo Hirano, Naoya Oribe, Takefumi Ueno, Yoji Hirano, Itta Nakamura, Tomofumi Miura, Shigenobu Kanba

**Affiliations:** 1 Department of Neuropsychiatry, Graduate School of Medical Sciences, Kyushu University, Fukuoka, Japan; 2 Clinical Neuroscience Division, Laboratory of Neuroscience, Department of Psychiatry, Boston VA Healthcare System, Brockton Division and Harvard Medical School, Brockton, Massachusetts, United States of America; Chiba University Center for Forensic Mental Health, Japan

## Abstract

Periodic auditory click stimulation has been reported to elicit an auditory steady state response (ASSR). The ASSR has been suggested to reflect the efficiency of γ-amino butyric acid (GABA) inhibitory interneuronal activity. Although a potential role for GABAergic dysfunction has been previously proposed, the role of neural synchronization in the ASSR in people with bipolar disorder (BD) has received little attention. In the current study, we investigated ASSRs to 20 Hz, 30 Hz, 40 Hz and 80 Hz click trains in BD patients. A total of 14 (4 males) BD patients and 25 (10 males) healthy controls participated in this study. ASSRs were obtained using whole-head 306-channel magnetoencephalography to calculate, ASSR power values and phase locking factors (PLF). BD patients exhibited significantly reduced mean ASSR power and PLF values bilaterally at frequencies of 30, 40, and 80 Hz (*p*<0.05 for these frequencies). At 20 Hz, bipolar patients showed no significant reduction in mean ASSR power and PLF values. There was a significant negative correlation between 80 Hz-ASSR-power values obtained from the right hemisphere and scores on the Hamilton Depression Rating Scale (rho = −0.86, *p = *0.0003). The current study showed reduced low and high gamma band ASSR power and PLF bilaterally with no significant beta band ASSR reduction in BD patients. BD patients are characterized by deficits in gamma band oscillations, which may be associated with GABA inhibitory interneuronal activity dysfunction.

## Introduction

Periodic auditory click stimulation elicits an auditory steady state response (ASSR) that synchronizes to both the phase and frequency of the click stimulus. Several magnetoencephalography (MEG) studies have reported that source generators of ASSR are restricted to the primary auditory cortex [1], [2]. Neural circuitry functioning in the primary auditory cortex can be assessed using MEG-ASSR. The ASSR can reveal information about neural activity with respect to phase synchronization and response magnitude. The ASSR exhibits resonant frequencies in response to click trains at approximately 40 Hz and 80 Hz, although 40 Hz click trains produce responses of a larger magnitude [3].

Responses between 14 and 30 Hz are categorized as beta band activity, and rhythms >30 Hz are categorized as gamma band activity [4]. In addition, gamma band activity is subdivided into low (30–70 Hz) and high gamma band (>70 Hz) oscillations [5]. It has been suggested that the ASSR reflects the efficiency of γ-amino butyric acid (GABA) inhibitory interneuronal activity, which control the timing of pyramidal neurons in layer II/III of the cortex [6], [7]. Additionally, interactions between pyramidal neurons and inhibitory neurons have been found to produce emergent oscillations [8]. Emrich et al. proposed that GABAergic dysfunction plays a role in bipolar disorder (BD), based on the efficacy of valproate in the treatment of patients with this disorder [9]. Moreover, a post-mortem study of BD patients reported down-regulation in the expression of GABAergic genes (e.g., glutamic acid decarboxylase) [10]. Since ASSR is linked to GABA activity, investigations of ASSR are important in understanding BD.

In an MEG study of ASSR in BD, Maharajh et al. reported that patients exhibited a reduced right 40-Hz ASSR [11]. An electroencephalography (EEG) study by O’Donnell et al. reported reduced 20-, 30-, 40-, and 50-Hz ASSR in BD patients [12]. In addition, Rass et al. reported reduced ASSR power at 40 Hz and reduced ASSR synchronization at 40 Hz- and 50 Hz- stimulation in BD patients [13]. Studies of ASSR in schizophrenia (SZ) have consistently reported reduced gamma band ASSR [14]–[18]. For example, Light et al. reported that SZ patients exhibited reductions in both the evoked power and phase synchronization of ASSR to 30- and 40- Hz stimulation, but exhibited normal responses to 20- Hz stimulation [16]. Uhlhaas et al. suggested that GABA is involved in the generation and synchronization of beta and gamma oscillations [4]. One computational modeling study (assuming that reduction of GABAergic interneurons increases the variability of GABA time constants) showed reduced 40 Hz responses and increased 20 Hz responses [19].

As discussed above, BD and SZ patients show similar patterns of ASSR deficits. Moreover, a post-mortem study reported a reduction in the numerical density of inhibitory interneurons in both BD and SZ [20]. Taken together, these findings indicate that neural circuitry dysfunction may exhibit similarities between these disorders at least to some extent. Recently, high gamma band oscillations have become a subject of increasing research interest [21], [22]. However, to our knowledge, only two studies have examined high gamma band ASSR (i.e., ASSR to 80 Hz click trains) in SZ [17], [18], with no studies of high gamma band ASSR in patients with BD. Overall, ASSR has received less attention in BD than in SZ research.

The current study used MEG to examine beta (ASSR to 20 Hz click trains), low (ASSR to 30 and 40 Hz click trains) and high gamma ASSR in BD patients. The present study was designed to test the hypothesis that BD patients exhibit reduced low and high gamma ASSR and no significant beta ASSR reduction.

## Results

### Demographic Characteristics

There were no significant group differences in age, handedness, self or parental SES or years of education (Table 1). There was no significant correlation between the dose of neuroleptic medication or lithium and ASSR power or PLF (−0.48≤rho≤0.63, 0.06≤*p*≤0.97 for neuroleptics; −0.65≤rho≤0.35, 0.08≤*p≤*1.0 for lithium). ASSR variables did not correlate significantly with valproate dosage, with the exception of significant negative correlations between right hemisphere 40 Hz-ASSR and the dosage (rho = −0.75, *p* = 0.02 for PLF; rho = −0.66, *p* = 0.05 for power).

**Table 1:** Demographic and Clinical Characteristics of Participants.

	HC	BD	df	*t* or *x^2^*	*p*
Sex, M/F, No	10/15	4/10	1	0.51	0.50
Age (years)	37.6±15.8	40.8±13.0	37	−0.68	0.50
Handedness	96.4±7.1	96.4±9.5	37	−0.01	0.99
SES	2.3±0.7	2.6±1.1	37	−0.85	0.40
Parental SES	2.8±1.0	3.1±1.1	37	−0.65	0.52
Education (years)	14.5±2.1	13.6±2.3	37	1.2	0.22
Symptom onset(years)		28.6±13.8			
Duration of illness(years)		11.6±9.9			
Medication dose(CPZ equiv., mg)		314±201			
YMRS		1.9±3.9			
SIGH-D		8.6±5.0			

Values are mean ± SD unless otherwise noted. HC: healthy controls, BD: patients with bipolar disorder,

SES = socioeconomic status, YMRS = Young Mania Rating Scale, SIGH-D = Structured Interview Guide for the Hamilton Depression Rating Scale.

Patients with BD were administered the following medications : N = 2 lithium & valproate; N = 1 lithium, quetiapine & zotepine; N = 1 lithium & quetiapine; N = 1 quetiapine, amoxapine & paroxetine; N = 1 valproate, amoxapine, trazodone & paroxetine; N = 1 valproate & quetiapine, N = 1 quetiapine & paroxetine; N = 1 lithium, valproate, quetiapine & amitriptyline; N = 1 valproate & trazodone; N = 1 lithium, valproate, olanzapine & risperidone; N = 1 valproate & quetiapine; N = 1 lithium, valproate & quetiapine; N = 1 lithium, quetiapine & levomepromazine.

To exclude the effects of transient gamma band responses [9], we also performed the analyses of the ASSR using a 200–500 ms window. The statistically significant results reported below remained the same. ASSR variables did not correlate significantly with demographic data or clinical scale scores (−0.008≤rho≤0.54, 0.07≤*p≤*0.93) in either group, with the exception of a significant negative correlation between right hemisphere 80 Hz-ASSR-power and the Structured Interview Guide for the Hamilton Depression Rating Scale (SIGH-D) scores (rho = −0.86, *p* = 0.0003) in participants with BD.

### Mean ASSR Power

**Figure 1:**
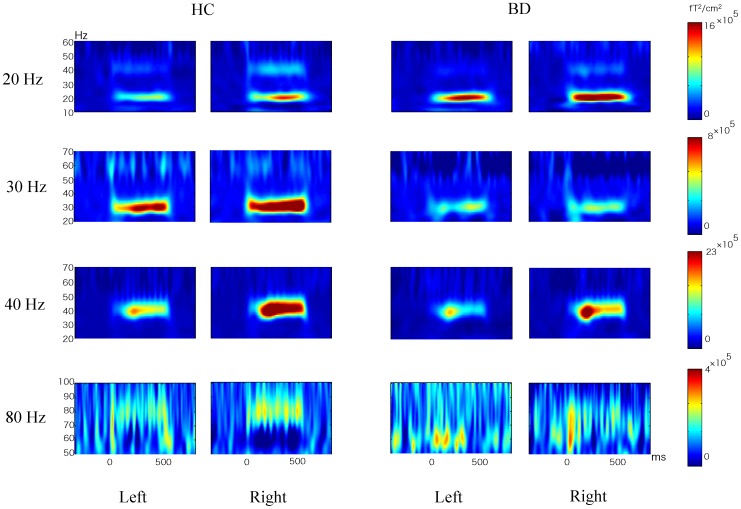
Group averaged time-frequency maps of ASSR-power for each hemisphere. The color scales signify ASSR-power. HC, healthy controls; BD, patients with bipolar disorder.

Figure 1 shows group-averaged time-frequency maps of ASSR power for each hemisphere. Values of mean±SD ASSR power are shown in Table 2. A repeated measures analysis of variance (ANOVA) demonstrated significant main effects of group (F [1], [37] = 7.0, *p = *0.01), frequency (F [3], [35] = 34.7, *p<*0.0001), and hemisphere (F [1], [37] = 10.9, *p = *0.002), and significant frequency-by-group (F [3], [35] = 3.4, *p = *0.03) and frequency-by-hemisphere (F [3], [35] = 3.4, *p = *0.03) interactions, with no other significant interactions (0.50≤*p≤*0.64). To delineate the significant frequency-by-group interaction, group differences were compared with *t*-tests using the average of both hemispheres for each frequency. Participants with BD showed significantly reduced ASSR power at 30-Hz (*t* [37] = 3.1, *p = *0.004), 40-Hz (*t* [37] = 2.6, *p = *0.01), and 80-Hz (*t* [37] = 2.2, *p = *0.03), while no significant group differences were observed at 20-Hz (*t* [37] = 0.38, *p = *0.71).

**Table 2:** Mean ASSR-power.

		HC (n = 25)(fT/cm)	BD (n = 14)(fT/cm)	df	*t*	*p*
20 Hz	Left	253.9±162.1	220.0±235.8	37	0.53	0.6
	Right	285.4±193.2	272.6±252.0	37	0.18	0.86
30 Hz	Left	264.4±176.0	152.5±80.1	37	2.2	0.03
	Right	318.3±187.0	166.8±112.2	37	3.2	0.003
40 Hz	Left	505.8±299.7	292.1±240.9	37	2.3	0.028
	Right	625.1±302.2	370.5±275.3	37	2.6	0.013
80 Hz	Left	76.6±73.5	46.1±35.7	37	1.7	0.09
	Right	96.5±84.8	48.9±39.7	37	2.4	0.023

Data are given as mean ± SD.

ASSR: auditory steady state response, HC: healthy controls,

BD: patients with bipolar disorder.

For 40 Hz harmonic response to 20 Hz stimulation, a repeated measures ANOVA demonstrated a significant main effect of hemisphere (F[1], [37] = 4.91, *p* = 0.033), a trend-level significant group effect (F[1], [37] = 2.91, *p* = 0.096), and no significant hemisphere-by-group interaction (F[1], [37] = 1.69, *p* = 0.20), indicating trend-level reductions of 40 Hz harmonic powers to 20 Hz stimulation in BD patients.

### Mean ASSR PLF

**Figure 2:**
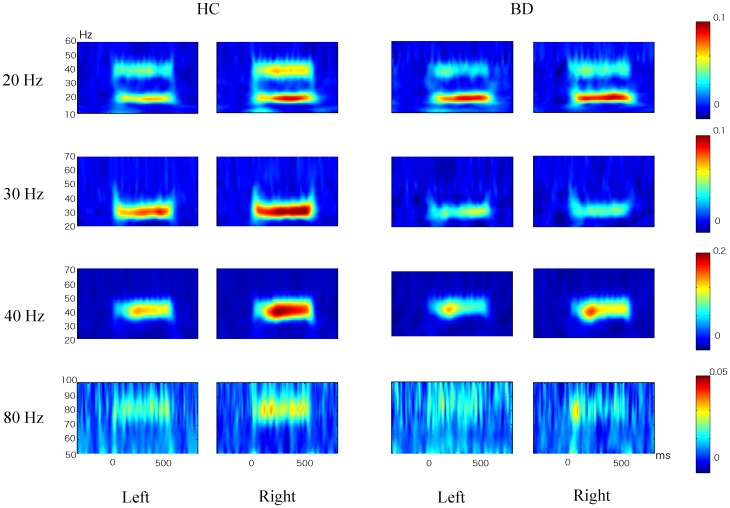
Group averaged time-frequency maps of ASSR-PLF for each hemisphere. The color scales signify ASSR-PLF value. HC, healthy controls; BD, patients with bipolar disorder.

Figure 2 shows group averaged time-frequency maps of ASSR PLF for each hemisphere. The mean±SD of ASSR PLF values are shown in Table 3. A repeated measures ANOVA demonstrated significant main effects of group (F [1], [37] = 12.0, *p = *0.001), frequency (F [3], [35] = 49.8, *p<*0.0001), and hemisphere (F [1], [37] = 10.7, *p = *0.002), and significant frequency-by-group (F [3], [35] = 4.3, *p = *0.02) and frequency-by-hemisphere (F [3], [35] = 6.3, *p = *0.002) interactions, with no other significant interactions (0.15≤*p*≤0.62). To delineate the significant frequency-by-group interaction, group differences were compared with *t*-tests, using the average of both hemispheres for each frequency. Participants with BD exhibited significantly reduced ASSR PLF at 30-Hz (*t* [37] = 3.1, *p<*0.0001), 40-Hz (*t* [37] = 3.0, *p = *0.005), and 80-Hz (*t* [37] = 2.3, *p = *0.03), while no significant group differences were observed for 20-Hz (*t* [37] = 1.5, *p = *0.17).

**Table 3:** Mean ASSR PLF.

		HC (n = 25)	BD (n = 14)	df	*t*	*p*
20 Hz	Left	0.038±0.022	0.027±0.026	37	1.3	0.2
	Right	0.043±0.026	0.031±0.028	37	1.4	0.17
30 Hz	Left	0.044±0.031	0.022±0.011	37	3.1	0.004
	Right	0.053±0.03	0.023±0.012	37	4.3	<0.001
40 Hz	Left	0.091±0.051	0.052±0.038	37	2.5	0.018
	Right	0.11±0.046	0.063±0.042	37	3.3	0.002
80 Hz	Left	0.013±0.011	0.008±0.007	37	1.3	0.188
	Right	0.016±0.011	0.007±0.006	37	2.6	0.013

Data are given as mean ± SD.

ASSR: auditory steady state response, PLF: phase locking factor,

HC: healthy controls, BD: patients with bipolar disorder.

For 40 Hz harmonic response to 20 Hz stimulation, a repeated measures ANOVA demonstrated no significant main effects of group (F[1], [37] = 2.33, *p* = 0.14) or hemisphere (F[1], [37] = 3.36, *p* = 0.075) and no significant hemisphere-by-group interaction (F[1], [37] = 1.21, p = 0.28).

### Dipole Moments and Locations

Table 4 shows the group mean dipole moments for each group. A repeated measures ANOVA demonstrated significant main effects of group (F [1], [37] = 18.9, *p = *0.03), frequency (F [3], [35] = 53.8, *p<*0.0001), and hemisphere (F [1], [37] = 8.8, *p = *0.005), and significant frequency-by-group (F [3], [35] = 4.9, *p = *0.003) interactions, with no other significant interactions (0.32≤*p≤*0.64). To delineate the significant frequency-by-group interaction, group differences were compared with *t*-tests using the average of both hemispheres for each frequency. Participants with BD showed significantly reduced dipole moments at 30-Hz (*t* [37] = 2.0, *p = *0.05), 40-Hz (*t* [37] = 3.1, *p = *0.003), and 80-Hz (*t* [37] = 2.0, *p = *0.05), while no significant group differences were observed for 20-Hz (*t* [37] =  −0.66, *p = *0.51).

**Table 4:** Dipole moments of the ASSR.

		HC (n = 25)	BD (n = 14)	df	*t*	*p*
		(nA/m)	(nA/m)			
20 Hz	Left	3.5±1.2	3.8±1.6	37	−0.55	0.59
	Right	3.6±1.4	3.9±1.3	37	−0.47	0.64
30 Hz	Left	2.9±1.1	2.5±1.1	37	1.1	0.29
	Right	3.9±1.4	2.9±0.9	37	2.4	0.02
40 Hz	Left	3.7±2.2	2.2±1.0	37	2.3	0.03
	Right	3.9±1.4	2.8±1.0	37	2.6	0.01
80 Hz	Left	1.3±0.8	1.1±0.3	37	1.0	0.31
	Right	1.6±0.9	1.2±0.4	37	1.5	0.14

Data are given as mean ± SD.

HC: healthy controls, BD: patients with bipolar disorder.

With respect to dipole locations, a multivariate ANOVA (MANOVA) demonstrated no group effect and no interactions related to group, indicating that there were no significant group differences for dipole locations of the ASSR (see Table 5).

**Table 5:** Dipole locations of the ASSR.

		Left (mm)			Right (mm)		
		x	y	z	x	y	z
20 Hz	HC (n = 25)	−45.6±4.9	6.6±12.7	61.6±9.0	49.1±7.3	9.5±9.5	61.4±12.8
	BD (n = 14)	−47.3±6.8	0.16±17.4	58.0±7.4	49.5±5.5	5.1±13.8	57.2±8.9
30 Hz	HC	−45.6±4.3	3.9±11.4	63.0±10.7	48.2±5.9	8.7±9.5	60.1±9.6
	BD	−45.8±5.1	3.0±16.4	60.9±8.0	47.5±7.8	5.7±14.7	59.4±8.0
40 Hz	HC	−47.0±5.2	4.3±13.0	60.9±10.9	50.6±11.8	9.3±10.5	58.3±14.3
	BD	−49.3±7.7	2.2±16.0	52.4±16.2	51.2±6.9	2.7±15.4	56.8±7.3
80 Hz	HC	−48.3±6.5	3.0±14.2	51.9±15.8	51.9±7.1	5.6±16.2	54.4±12.3
	BD	−46.7±5.4	−0.33±17.2	49.7±17.6	50.7±6.1	5.8±11.9	44.1±20.3

Data are given as mean ± SD. HC: healthy controls, BD: patients with bipolar disorder.

The zero point was the mid-point of the line connecting the bilateral preauricular points. The x-axis was the line from the left to the right with positive values toward the right, the y-axis was the postero-anterior line with positive values presented anteriorly, and the z-axis was the ventro-dorsal line with positive values located dorsally.

## Discussion

The current study investigated the MEG-ASSR elicited by click trains of 20, 30, 40 and 80 Hz, and symptom-ASSR associations in patients with BD. The major findings in this study were: [1] BD patients exhibited bilaterally reduced mean ASSR power and PLF to 30-, 40- and 80- Hz stimulation, with no significant reduction to 20- Hz stimulation; [2] there was a significant negative correlation between right hemisphere 80 Hz-ASSR-power values and SIGH-D scores in patients with BD; [3] No significant group differences were observed in the dipole locations of ASSR.

To our knowledge, this is the first study to demonstrate both high and low gamma band ASSR deficits in patients with BD. Previous EEG studies reported reduced 20-, 30-, 40-, and 50-Hz ASSR in people with BD [12] and reduced ASSR at 30 and 40 Hz in people with psychotic BD [23]. Rass et al. reported reduced ASSR power at 40 Hz and reduced ASSR synchronization to 40 Hz- and 50 Hz- stimulation in BD patients [13]. One MEG study reported that patients exhibited reduced right ASSR to 40 Hz- stimulation [11]. The present results partially support these previous findings. For the high gamma band, oscillations can be useful markers of cortical activity during a variety of cognitive tasks [21] and may reflect a fundamental aspect of temporal coding in cortical networks [22]. Additionally, different functions between beta and gamma oscillations have been suggested. Beta oscillations are related to sensory gating, attention and perception, and gamma oscillations are associated with memory and consciousness as well as attention and perception [4]. Future studies should investigate the relationship between ASSRs and neural oscillatory activities during cognitive tasks in patients with BD, to clarify ASSR-cognitive related oscillations.

It has been suggested that GABAergic dysfunction plays a role in BD patients [9], [10]. The administration of mood stabilizers, such as valproate, carbamazepine, lithium, and lamotrigine, has been reported to increase GABA turnover in the mouse and rat brain [24]–[27]. In addition, valproate has been shown to increase plasma GABA levels in humans, suggesting that it enhances GABA activity in the human brain [28]–[30]. Recent *in vitro* studies have suggested that beta2 (20–30 Hz) oscillations are different from gamma oscillations in terms of generation. For instance, Cunningham et al. reported that the fast rhythmic bursting neurons in layer II/III play a crucial role in the generation of gamma oscillations [31]. GABAergic neurons have been reported to play a crucial role in the primary generation of gamma oscillations and their local synchronization [32]. In addition, direct electronic coupling through gap junctions between inhibitory neurons also contributes to the synchronization of gamma oscillations [33]. Both low and high gamma band oscillations can be generated by recurrent inhibition, but differ in their relationship to the spiking activity of parvalbumin-containing interneurons; in terms of their pharmacological modulation profiles as well as their layer specificity [5]. Conversely, an *in vitro* study by Roopun et al. reported that beta2 oscillations occurred in layer V pyramidal cells [34]. Moreover, this study indicated that beta2 oscillations are involved in gap junctional coupling and are independent of chemical synaptic transmission. The present study reported gamma band ASSR reduction and no significant reduction of beta band ASSR in BD patients, suggesting that BD might be characterized by hypofunction of GABA interneurons related to the fast rhythmic bursting neurons in layer II/III.

The present results revealed a significant negative correlation between right hemisphere 80 Hz-ASSR-power values and SIGH-D scores, indicating that BD patients with more severe depressive symptoms exhibited more reduced 80 Hz-ASSR power in the right hemisphere. However, this correlation should be confirmed in a larger sample. Rass et al. recorded 20-, 30-, 40-, and 50-Hz ASSRs in BD, and investigated associations between ASSRs and clinical status, cognitive function, and pharmacological treatment [13]. They reported that BD patients taking psychotropic medication exhibited decreased PLF relative to BD patients who had withdrawn from medication. In this study, mood state, psychotic features, cognitive performance, smoking, or history of substance use disorder were unrelated to ASSRs. Future studies that incorporate an assessment of patients before and after medication would be helpful in clarifying the associations between clinical symptoms and ASSR deficits in people with BD.

Reite et al. investigated ASSR source locations in people with BD. In normal control subjects the right hemisphere source was superior to the left, but no such hemisphere asymmetry was observed in BD patients [1]. However, the present results revealed no significant group differences in the dipole locations of ASSR. The heterogeneity of BD patients may account for this discrepancy. For example, the BD patients in the present study had never experienced psychotic symptoms and the sample was predominantly female, while Reite et al. examined 10 individuals with BD who had a history of psychosis and seven with no history of psychosis [1]. The ASSR of BD patients with a history of psychosis requires further investigation.

Several potential limitations of the current study should be considered. We were unable to exclude any treatment effects of mood stabilizers, neuroleptics or antidepressants on ASSR abnormalities in BD patients, and we found significant negative correlations between right hemisphere 40 Hz-ASSR and valproate dosage. Cross-sectional studies with more homogenous patient groups (drug-free vs medicated), as well as studies that assess participants before and after treatment with specific medications (thus controlling for health status) are required in future. Moreover, the effects of gender, and the ASSR of BD patients with a history of psychosis require further investigation.

Overall, the current study showed that BD patients exhibit reduced low and high gamma ASSR power and PLF bilaterally, with no significant beta band ASSR reduction. BD is characterized by gamma band ASSR deficits, which may be associated with dysfunctions of GABA inhibitory interneuronal activity.

## Materials and Methods

### Subjects

MEG data obtained from 14 (4 males, 10 females) individuals with BD and 25 (10 males, 15 females) healthy controls (HC) were analyzed in the present study. The data from 22 of the 25 HC participants were analyzed in our previous study [18]. The data from 14 BD and 3 HC participants were newly recorded and analyzed for the present study. MEG recording was conducted between September 2007 and December 2009 for the HC group, and from July 2007 to May 2010 for the BD patients. We used the same recording equipment for both groups. All participants had normal hearing, were aged 20–60 years and were right-handed [assessed via Edinburgh Inventory [35]]. After being given a complete description of the study, all participants gave written informed consent in accord with the regulations of the Ethics Committee of the Graduate School of Medical Sciences, Kyushu University. Two senior clinical psychiatrists confirmed that all subjects had the ability to consent to participate in the examination. The exclusion criteria were: 1) neurological illness or major head trauma that would result in abnormal electroencephalography; 2) electroconvulsive therapy; 3) alcohol or drug dependence; 4) alcohol or drug abuse within the past five years; or 5) a verbal intelligence quotient below 75. HCs were screened using the Structured Clinical Interview (SCID), non-patient edition. No HCs exhibited any Axis-I psychiatric disorders, nor did their first-degree relatives.

**Figure 3:**
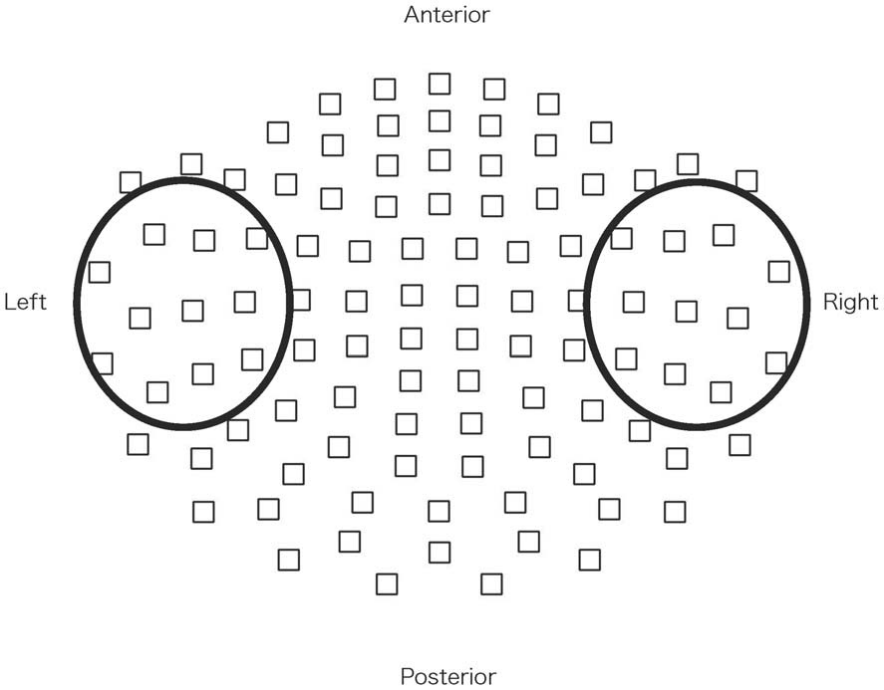
Layout of the measured channels. The MEG signals were acquired using a whole-head, 306-channel sensor array comprised of 102 identical triple-sensor elements. Each sensor consisted of two orthogonal planar-type gradiometers and one magnetometer. We used 11 sensors (a 22-channel orthogonal gradiometer) around the location that elicited the strongest response in each hemisphere. Circled squares indicate the sensors used for analysis.

All patients were recruited from Kyushu University Hospital and were diagnosed based on the SCID-DSM IV and medical records. No BD patients exhibited psychotic episodes. The patients were assessed using the Young Mania Rating Scale (YMRS) [36] and SIGH-D [37]. Demographic data for all subjects are presented in Table 1. Based on the criteria for depression [38] and euthymia [39], seven patients showed mild depression and seven were euthymic. Eight patients were receiving neuroleptic medication [typical neuroleptics (1/8 patients), atypical (7/8)], with a mean daily dose equivalent to 314±201 mg of chlorpromazine [40]. Regarding mood stabilizers, lithium was administered with a mean daily dose of 750±141 mg in eight BD patients, and valproate was administered with a mean daily dose of 844±445 mg in nine BD patients. The footnote in Table 1 lists the patients’ medication.

### Stimuli

The stimuli consisted of 1-msec clicking sounds, presented binaurally as trains of clicks for each stimulus frequency (20, 30, 40 and 80 Hz). The duration of each click train was 500 msec, and the intensity of the click trains was 80 dB sound pressure level. The inter-train interval was 500 msec. The mean number of presented click trains in one block was 313.9±105.7 for HC and 306.4±60.4 for BD, and there was no significant group difference (*t* [37] = 0.24, *p* = 0.81). The order of blocks was randomized across subjects.

### Data Acquisition and Processing

The MEG signals were acquired using a whole-head, 306-channel sensor array (Vectorview; ELEKTA Neuromag, Helsinki, Finland). In this study, we analyzed MEG data recorded from 22-channel, planar-type gradiometers located at the sensor exhibiting the strongest response. This procedure was conducted for each hemisphere (Figure 3) based on our previous methods [18]. Prior to recording, four head position indicator (HPI) coils were attached to the scalp, and a three-dimensional (3D) digitizer was used to measure the anatomical landmarks of the head with respect to the HPI coils. The precise location of the head with respect to the sensor array was determined using the HPI coils. A band pass filter for recording was set to 0.01–330 Hz, and the sampling rate was 1 kHz. The subjects were instructed to keep their eyes open, remain attentive and listen to the trains of clicks presented through earphones. A spatio-temporal signal space separation (tSSS) method was applied off-line to the recorded raw data [41]. tSSS-reconstructed raw data with signal variations exceeding 4000 fT were excluded, and 200 responses were averaged for each type of stimulus as a result. The data were averaged with the following conditions: the analyzed period included the duration 400 ms before and 900 ms after stimulus onset.

### Frequency Analysis

We used an estimation of the time-frequency energy based on the wavelet transform of the signal. The signal was convoluted by complex Morlet wavelets 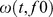 having a Gaussian shape with the wavelet being centered at the center frequency 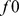 and time 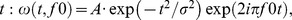 where 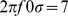 in 1-Hz steps. Wavelets were normalized so that their total energy was 1, with the normalization factor *^A^* equal to 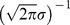. We defined the squared modulus of the result of the convolution of a complex wavelet 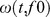 with the averaged responses 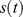: power 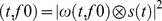 as the ASSR-power, where the symbol 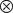 indicates convolution. The square-root transform was applied to the ASSR-power for normalization. We also calculated the ASSR-phase-locking factor (PLF) using the following formula: PLF 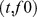 = 
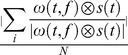
. The PLF ranges from 0 (purely non-phase-locked activity) to 1 (strictly phase-locked activity). In calculating the power and PLF, we applied a baseline correction (from −200 to −100 msec). The mean power and PLF from 0–500 msec for each stimulus were averaged across 10-Hz bands.

### Dipole Moments and Source Localization

The averaged responses were digitally filtered using a Butterworth filter (band pass; 15–25 Hz for the 20 Hz stimulation, 25–35 Hz for 30 Hz, 35–45 Hz for 40 Hz, and 75–85 Hz for 80 Hz). A single moving equivalent current dipole source model was applied, and dipole fits in each hemisphere were calculated by a least-squares fit. Single source dipole localization was performed for each time-point for 0–500 msec after stimulus onset. Only dipoles with goodness-of-fit criteria (>0.9) were chosen. The dipole locations were expressed by x, y, and z-coordinates.

### Statistical Analysis

The mean ASSR powers and PLF were analyzed using a repeated measures ANOVA with group (BD or HC) as a between-subjects factor, and frequency (20, 30, 40 or 80 Hz) and hemisphere (left or right) as within-subjects factors. When significant interactions involving the group factor were identified, *post-hoc* analyses were conducted using t-tests. Additionally, 40 Hz harmonic ASSR powers and PLF to 20 Hz stimulation were analyzed using a repeated measures ANOVA with group as a between-subjects factor, and hemisphere as a within-subjects factor. For dipole locations, MANOVA was performed with group as a between-subjects factor, and frequency, hemisphere and axis (x, y or z) as within-subjects factors. Degrees of freedom were adjusted with the Huynh-Feldt epsilon for factors with more than two levels. Spearman’s rho was used for correlation analyses. All results were considered significant at *p≤*0.05.
